# Identification of Viruses in Cases of Pediatric Acute Encephalitis and Encephalopathy Using Next-Generation Sequencing

**DOI:** 10.1038/srep33452

**Published:** 2016-09-14

**Authors:** Jun-ichi Kawada, Yusuke Okuno, Yuka Torii, Ryo Okada, Satoshi Hayano, Shotaro Ando, Yasuko Kamiya, Seiji Kojima, Yoshinori Ito

**Affiliations:** 1 Departments of Pediatrics, Nagoya University Graduate School of Medicine, 65 Tsurumai-cho, Showa-ku, Nagoya 466-8550, Japan; 2 Horticultural Research Institute, Ibaraki Agricultural Center, 3165-1 Ago, Kasama, 319-0292, Japan

## Abstract

Acute encephalitis/encephalopathy is a severe neurological syndrome that is occasionally associated with viral infection. Comprehensive virus detection assays are desirable because viral pathogens have not been identified in many cases. We evaluated the utility of next-generation sequencing (NGS) for detecting viruses in clinical samples of encephalitis/encephalopathy patients. We first determined the sensitivity and quantitative performance of NGS by comparing the NGS-determined number of sequences of human herpesvirus-6 (HHV-6) in clinical serum samples with the HHV-6 load measured using real-time PCR. HHV-6 was measured as it occasionally causes neurologic disorders in children. The sensitivity of NGS for detection of HHV-6 sequences was equivalent to that of real-time PCR, and the number of HHV-6 reads was significantly correlated with HHV-6 load. Next, we investigated the ability of NGS to detect viral sequences in 18 pediatric patients with acute encephalitis/encephalopathy of unknown etiology. A large number of Coxsackievirus A9 and mumps viral sequences were detected in the cerebrospinal fluid of 2 and 1 patients, respectively. In addition, Torque teno virus and Pepper mild mottle viral sequences were detected in the sera of one patient each. These data indicate that NGS is useful for detection of causative viruses in patients with pediatric encephalitis/encephalopathy.

Acute encephalitis and encephalopathy are characterized by an altered level of consciousness or status epilepticus convulsions within a few days of developing a high-grade fever and are associated with high morbidity and mortality. Patients with evidence of intracerebral inflammation are diagnosed with encephalitis, but distinguishing encephalitis from encephalopathy is sometimes difficult in the clinical setting. The most commonly demonstrated pathogenic mechanism of acute encephalitis/encephalopathy is infection, and viruses such as herpes simplex virus (HSV), influenza virus, and human herpesvirus 6 (HHV-6) are important known etiologic agents in pediatric patients^1^,^2^. To identify the viral pathogen of encephalitis/encephalopathy, PCR and viral antigen detection kits have been used for some specific viruses. On the other hand, the pathogens of acute encephalitis/encephalopathy have not been identified in a large proportion of cases because detection assays have not been established or are not commonly available for many viruses. For example, the Californian Encephalitis Project, which included 1570 patients, failed to identify cases of encephalitis in 63% of patients^1^. However, identification of causative agents is important for the correct diagnosis and may be necessary for selection of a specific treatment.

Next-generation sequencing (NGS) offers the prospect of relatively unbiased testing for all previously catalogued and sequenced viruses in a single test. Where conventional viral detection assays, such as serology, virus isolation, and PCR focus on a defined set of candidate viruses, NGS provides an unbiased survey of DNA or RNA sequences present in a sample. Recently, NGS has been applied for detection of viruses and discovery of novel viruses in clinical samples^3^,^4^. Although identification of the viral pathogen in encephalitis/encephalopathy patients has been challenging, several studies have shown that NGS is useful for identification of viral pathogens by detecting virus-driven DNA or RNA sequences present in cerebrospinal fluid (CSF) or brain tissue^5^,^6^. In this study, we investigated CSF and serum samples from pediatric acute encephalitis/encephalopathy patients using NGS.

## Results

### Identification of viral sequences in clinical samples using NGS

**Figure 1:**
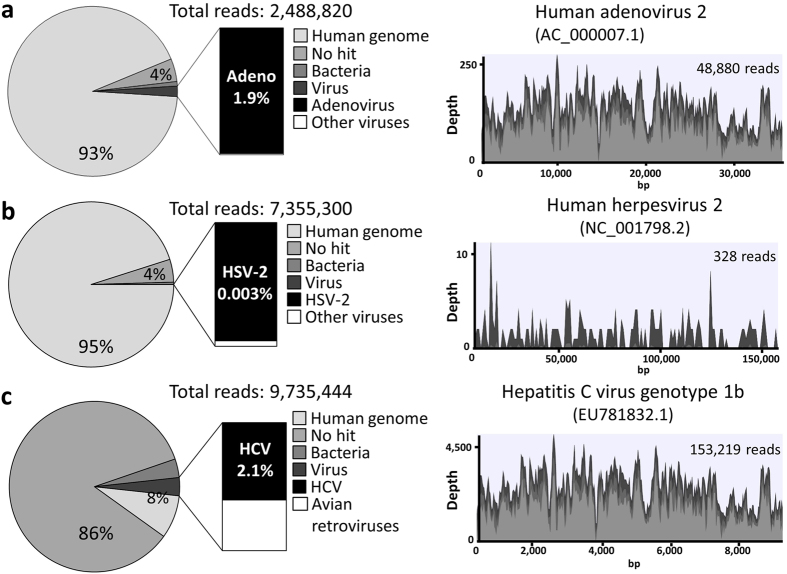
Identification of viral sequences in clinical samples using next-generation sequencing. Pie charts show classification of reads from each library into human, unknown categories (no hit), bacteria and viruses. Total reads were mapped against the reference genomes using the CLC Genomics Workbench. Light gray, gray, and very light gray colors in the viral genome alignments represent minimal, average, and maximal coverage in the aggregated 100 bp (human adenovirus and hepatitis C virus) or 1 kbp (human herpesvirus 2) region, respectively. (**a**) Sequencing results of the DNA library prepared from the serum of a patient with adenovirus fulminant hepatitis. Sequencing reads were mapped to the reference genome of human adenovirus 2. (**b**) Sequencing results of the DNA library prepared from cerebrospinal fluid of a patient with neonatal herpes encephalitis. Sequencing reads were mapped to the reference genome of human herpesvirus 2. (**c**) Sequencing results of the RNA library prepared from the serum of a patient with hepatitis C virus infection. Sequencing reads were mapped to the reference genome of Hepatitis C virus genotype 1b.

To validate the NGS-based approach to detect virus-derived sequences in clinical samples, serum or CSF samples obtained from patients with a defined diagnosis of viral infection were examined. Representative results of patients with adenovirus fulminant hepatitis (serum), HSV encephalitis (CSF), and HCV (serum) are shown in Fig. 1. The patient with adenovirus fulminant hepatitis was a 12-year-old female with acute myeloid leukemia who had received a bone marrow transplantation. She developed hepatitis on day 103 after transplantation, and died of hepatic failure on day 113. The details of this case were reported previously^7^. The patient with HSV encephalitis was a 12-day-old male newborn who presented with fever and seizure. He was diagnosed with neonatal HSV encephalitis by real-time PCR of CSF. The patient with HCV infection was a 14-year-old male who was infected by perinatal transmission. His liver function tests have been normal, but high HCV load was detected.

With DNA sequencing, human sequences were the most abundant, and the vast majority of detected viral sequences corresponded to a single predominant virus, as expected (Fig. 1a,b). Nearly complete adenoviral genomes were detected with high coverage, and sequencing results of the hexon gene indicated that the detected adenovirus was classified into type 2, which was consistent with the results of virus isolation (data not shown). On the other hand, with sequencing of RNA in the serum of the HCV patient, HCV sequences represented 2.1% of total reads (Fig. 1c). Sequencing results of the NS5B region indicated that the detected HCV was classified into genotype 1b (data not shown). However, unclassified sequences derived from Illumina adaptor sequences were the most abundant due to the low amount of input RNA for library preparation and subsequent preferential formation of adaptor dimers. Prior filtering of such non-essential artifacts decreases the heavy computational burden of BLAST search. Importantly, all RNA libraries contained sequences mapping to avian retroviral reference genomes such as avian myeloblastosis-associated virus type 1 and Rous sarcoma virus with high coverage (data not shown). However, they were likely derived from residual avian retroviral genomic material in the ScriptSeq reverse transcriptase enzyme (Illumina, San Diego, CA)^8^. For the viral metagenomics analysis using NGS, it has been shown that the ScriptSeq library preparation method retrieved more virus reads than TruSeq RNA Kit v2 (Illumina), but avian retroviral sequences were detected only in the libraries prepared with ScriptSeq kit^9^.

### Correlation between results from NGS and real-time PCR

**Figure 2:**
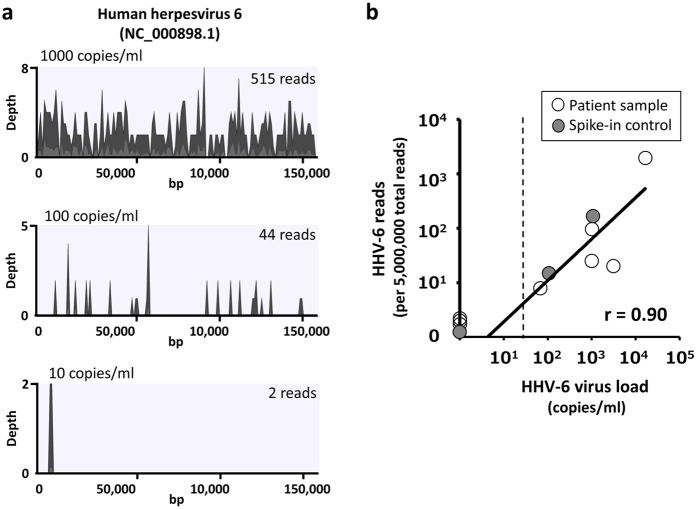
Correlation between the results of next-generation sequencing and real-time PCR. (**a**) DNA libraries were prepared from sera spiked with different loads of human herpesvirus 6 (HHV-6), and sequencing reads were mapped to the reference genome. Gray and very light gray colors in the viral genome alignments represent average and maximal coverage in aggregated 1 kbp region. The number of HHV-6 reads is indicated in the upper right of each coverage plot. Total sequencing reads of sera spiked with 1000, 100, and 10 copies/ml HHV-6 are 15514298, 14904684, and 16868138 reads, respectively. (**b**) The number of HHV-6 reads detected by next-generation sequencing in sera was compared with the HHV-6 viral load that was measured with real-time PCR. Open circles indicate sera from patients with HHV-6 infection, and closed circles indicate sera spiked with HHV-6, as shown in Fig. 2a. The number of HHV-6 reads per sample is shown per 5,000,000 total reads. Correlations were determined using regression analysis. Dotted line, detection limit of real-time PCR.

First, we assessed the sensitivity of NGS for detection of viral sequences using sera spiked with HHV-6. Cell-free HHV-6 fluid was prepared by collecting supernatants from HHV-6 infected cord blood mononuclear cells. As shown in Fig. 2a, NGS was able to detect 44 and 2 reads of HHV-6 sequences in serum with 100 and 10 copies/ml of HHV-6, respectively. Considering that detection limit of real-time PCR for HHV-6 is around 50 copies/ml, the sensitivity of the NGS-based approach for detection of HHV-6 was equivalent to that of real-time PCR. To confirm the quantitative performance of NGS, the number of HHV-6 sequences detected in sera of patients was compared with the HHV-6 load measured using real-time PCR. The number of HHV-6 sequences per sample was set to per 5,000,000 total reads because the sensitivity of NGS is dependent on sequencing depth. As shown in Fig. 2b, the number of HHV-6 reads was significantly correlated with HHV-6 load (r = 0.90, *p* < 0.01). These results suggest that the sensitivity and quantitative performance of the NGS-based approach for detection of DNA viral sequences was equivalent to that of real-time PCR in cell-free clinical samples with a sequencing depth of 5,000,000 reads.

### Detection of viral sequences in clinical samples of acute encephalitis/encephalopathy

**Figure 3:**
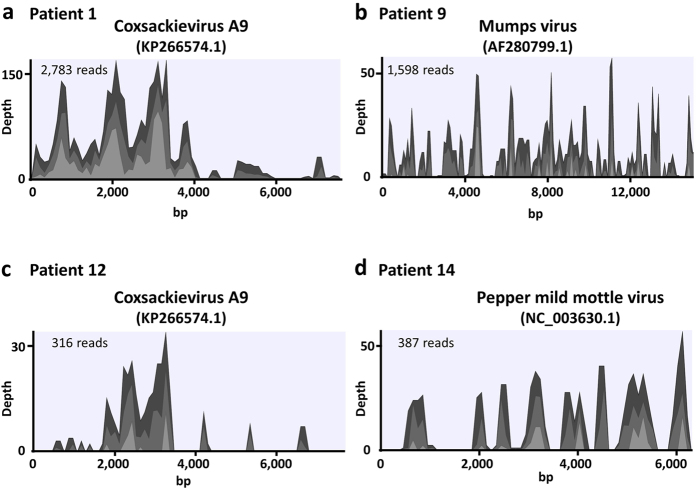
Coverage plot of viral genomes. Sequencing reads detected in each patient were mapped to the reference genome of Coxsackievirus A9 (**a**,**c**), mumps (**b**), and Pepper mild mottle virus (**d**). Light gray, gray, and very light gray colors in the viral genome alignments represent minimal, average, and maximal coverage in aggregated 100 bp region, respectively.

**Figure 4:**
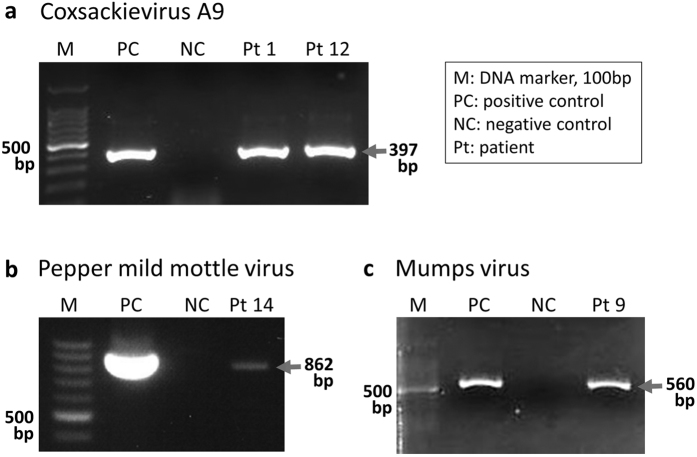
Amplification of virus RNA from clinical samples. Coxsackievirus A9 and mumps virus were PCR amplified in cerebrospinal fluid of patients 1 and 12, and in patient 9, respectively. Pepper mild mottle virus was PCR amplified in the serum of patient 14. Expected product size of each PCR is shown.

Next, we investigated the CSF and serum samples of patients with acute encephalitis/encephalopathy of unknown etiology. A summary of the patients and the viruses that were detected is shown in Table 1. In some CSF samples, DNA sequencing could not be performed because the concentration of the extracted DNA was not sufficient to prepare DNA libraries. Sequences that mapped to a specific viral genome (except avian retroviruses) with more than 10 reads were detected in three of 16 CSF samples and in two of 15 serum samples (Table 1). Substantial reads of Coxsackievirus A9 sequences were detected in the CSF of patients 1 and 12 (Fig. 3a,c). These sequences aligned with other enteroviral genomes such as Coxsackievirus B3 and Echovirus E30, whereas alignment with the VP1 region suggested that they were derived from Coxsackievirus A9 in both patients (data not shown). Furthermore, mumps viral sequences were detected in the CSF sample from patient 9, and these sequences covered the reference mumps genome (Fig. 3b). Alignment with the SH genomic region indicated that the detected mumps virus was genotype G and not a vaccine strain (data not shown). On the other hand, 13 reads of the Torque teno virus (TTV) and 298 reads of the Pepper mild mottle virus (PMMoV) were detected in the serum of patients 2 and 14, respectively. These virus-derived sequences were not detected in CSF. We confirmed that PPMoV-derived sequences covered the reference genome (Fig. 3d). The presence of Coxsackievirus A9 and mumps virus RNA in the CSF, and PMMoV RNA in the serum was confirmed by RT-PCR (Fig. 4).

In addition to viral sequences, a significant number of sequences derived from environmental bacteria such as Acinetobacteria and Proteobacteria were detected in all CSF and serum samples (data not shown). However, sequences of bacteria that have been recognized as causes of encephalitis, such as Listeria or *Coxiella burnetii*, were not detected.

## Discussion

In this study, we evaluated the utility of NGS for detecting viral sequences in CSF and serum and investigated the causative virus in 18 pediatric patients with acute encephalitis/encephalopathy. Sequences mapping to the Coxsackievirus A9 virus and the mumps viral genome were found in CSF, and these viruses were considered to be the causative viral pathogen of encephalitis/encephalopathy. Non-polio enteroviruses are a major cause of encephalitis in children, and Coxsackievirus A9 virus is one of the most frequent serotypes associated with encephalitis^10^. On the other hand, mumps is one of the most frequent causes of confirmed viral encephalitis in the pre-vaccine era. In Japan, outbreaks of mumps are not uncommon because universal vaccination against mumps has not been introduced. However, the patient with suspected mumps encephalitis in this study had received one dose of the mumps vaccine at the age of 1 year. Sequences detected in the CSF aligned with the wild-type mumps genome rather than vaccine strains, suggesting primary or secondary vaccine failure in this patient. These results suggest that the NGS-based approach for detection of virus-derived sequences in CSF may be a useful and reliable method for identification of the viral pathogen of encephalitis/encephalopathy.

On the other hand, no significant viral reads were detected in 13 of the 16 CSF samples. In general, free RNA from sera or CSF is degraded and obtained in low yield^11^,^12^. Extraction of RNA from CSF without pleocytosis was performed with carrier RNA to yield sufficient RNA for library preparation. However, no virus pathogens were detected in these CSF samples, suggesting that RNA sequencing of CSF specimens with very low RNA concentrations may not be very useful. Because of low RNA yield in CSF, the detection of virus-specific RNA has lower sensitivity for diagnosis than other methods. For example, virus RNA detection using RT-PCR in CSF of patients with West Nile virus encephalitis is less sensitive than detection of virus specific IgM antibody in CSF^13^. It is possible that some of the virus-specific IgM in CSF is more stable than virus RNA. Furthermore, we were unable to perform DNA sequencing in CSF samples without pleocytosis because DNA yields were not sufficient for DNA library preparation. However, it has been shown that RNA sequencing can be used to detect mRNA reads derived from DNA viruses^9^.

In addition to CSF, we investigated the sera from patients with encephalitis/encephalopathy because some viral pathogens such as HHV-6 are sometimes detected in sera but not in CSF^14^. Some cases of virus-associated encephalopathy may be a consequence of a systemic immune response rather than a result of direct viral invasion of the central nervous system^15^,^16^. Interestingly, sequences mapping to PMMoV were detected in the serum of one patient. PMMoV is a plant RNA virus of the genus *Tobamovirus*. Recently, metagenomics studies have identified PMMoV in the stool of healthy subjects^17^. Furthermore, the presence of PMMoV in stool is associated with seropositivity for PMMoV-IgM antibodies and clinical symptoms such as fever and abdominal pain^17^. On the other hand, detection of PMMoV in human peripheral blood has not been reported. Further investigations are required to clarify whether PMMoV is associated with human disease or is just an innocent bystander. A small number of TTV sequences were detected in the serum of another patient. TTV infections are frequent in humans, but a direct association between TTV and specific diseases has not been established because TTV is sometimes detected in apparently healthy individuals^18^.

As with previous studies of NGS-based pathogen analysis, a significant number of sequences derived from different types of bacteria such as Acinetobacteria and Proteobacteria were detected in all CSF and serum samples (data not shown)^19^,^20^. Although cultures of blood and CSF for bacteria were negative in all patients, some of these bacteria may have been causative agents of encephalitis/encephalopathy. Another possibility is that sequences of normal bacterial flora in humans may be persistently detected in the sera or CSF from patients without bacteremia or meningitis. However, significant levels of bacterial reads, which likely arise from environmental sources, are detected in DNA and RNA sequencing libraries prepared from clinical specimens or tissue culture cells^19^. For that reason, bacterial contamination is a relevant issue that needs to be extensively addressed before widespread use of NGS-based pathogen detection can be implemented. Distinguishing microbes of interest from contaminants, particularly bacteria, remains difficult.

In conclusion, we have shown that NGS is useful for detection of causative viruses in patients with pediatric encephalitis/encephalopathy. Although routine use of NGS may be difficult because it is costly and labor intense, an unbiased and highly sensitive NGS-based approach has great potential for analysis of pathogens present in clinical samples, and will likely contribute in clinical and public health settings.

## Methods

### Ethics Statement

The study design and methods were approved by the Institutional Review Board of Nagoya University Hospital (IRB number:5069). The methods were carried out in accordance with the approved guidelines. Informed consent was obtained from all patients or their guardians.

### Patients and samples

A total of 18 patients with pediatric acute encephalitis/encephalopathy of unknown etiology were enrolled in this study (Table 1). Acute encephalopathy/encephalitis was defined as occurring in patients with a depressed or altered level of consciousness lasting more than 24 hours and one or more of the following: fever (>38 °C) during the presenting illness; seizure(s) and/or focal neurological findings; CSF pleocytosis; abnormal results of an electroencephalogram; abnormal results of neuroimaging^1^. Cultures of blood and CSF for bacteria, rapid influenza test, and PCR for HSV, HHV-6, and HHV-7 were negative in all patients. RT-PCR for enterovirus was not performed at enrollment. Serum and CSF samples were obtained in the acute phase (within 2 days of the onset of neurologic involvement) and stored at −30 °C until use. Clinical samples obtained from patients with a defined diagnosis of viral infection such as adenovirus hepatitis, HSV encephalitis, hepatitis C virus (HCV), or HHV-6 encephalopathy were used to validate the NGS-based approach to detect virus-derived sequences. Real-time PCR of HHV-6 was performed with a QuantiTect multiplex PCR kit (Qiagen, Hilden, Germany) as described previously^21^, and primers and probes are shown in Table 2.

### Library preparation and sequencing

Samples were filtered through a 0.45-μm filter (Merck-Millipore, Temecula, CA) to remove blood cells and bacteria, and extraction of total nucleic acids was performed with a QIAamp UCP Pathogen Mini kit (Qiagen) in accordance with the manufacturer’s instructions. Extraction of RNA from CSF without pleocytosis was performed with a QIAamp Viral RNA Mini kit (Qiagen) with carrier RNA to yield sufficient RNA for library preparation. Concentrations of extracted DNA and RNA were measured using a Qubit assay kit (Thermo Fisher Scientific, Walthman, MA). Concentrations of DNA from sera and CSF with pleocytosis ranged from 0.1 to 8.2 ng/μl (median, 0.5 ng/μl). On the other hand, concentrations of DNA from CSF without pleocytosis, and RNA extracted without carrier RNA were below the detection limit (<0.01 ng/μl). We could not assess the quality of RNA because the amount of extracted RNA was very low. Before preparation of the RNA sequencing library, extracted nucleic acids were treated with Turbo DNase (Ambion, Darmstad, Germany) for 30 min at 37 °C to digest host DNA. DNA and RNA sequencing libraries were prepared using a Nextera XT DNA Sample preparation kit (Illumina) and ScriptSeq v2 (Illumina), respectively. Library quality was determined using an Agilent 2200 TapeStation (Agilent, Santa Clara, CA). Fragment sizes and concentrations of libraries ranged from 180 to 511 bp (median, 200 bp) and from 0.7 to 22.0 nM (median, 1.6 nM), respectively. Indexed samples were pooled and sequenced on MiSeq (Illumina) with 2 × 75 bp or HiSeq 2500 (Illumina) with the 2 × 150 bp pair-end protocol to obtain an average of 8,674,293 reads. Obtained short reads are deposited in the DNA Data bank of Japan (Accession number: DRA004741).

### Data processing

For identification of virus-derived sequences from NGS data, the cloud-computing pipeline, MePIC v2.0 (National Institute of Infectious Diseases, Japan) was used as follows^22^. Raw sequencing reads were processed by removing adaptors and low-quality sequences, followed by subtraction of human reads. For the remaining reads, the MEGABLAST program was used to search sequences for known nucleotide sequences in the database including viruses. To summarize the taxonomic information, the metagenome analyzer, MEGAN5 (University of Tübingen, Tübingen, Germany), was used^23^. Alignment with each viral reference genome was analyzed by CLC workbench (CLC bio; Qiagen). To avoid making calls based on potentially spurious alignments, detection of more than 10 reads of sequences mapping to the viral reference genome was considered to indicate the presence of the virus in this study.

### RT-PCR

RT-nested PCR of Coxsackievirus A9 and mumps virus as well as RT-PCR of PMMoV were performed as described previously with the modified primers shown in Table 2 24,25. One-step RT-PCR and second PCR was carried out using AccessQuick RT-PCR (Promega, Madison, WI) and Takara Ex Taq (Takara Bio, Kusatsu, Japan), respectively.

## Additional Information

**How to cite this article**: Kawada, J. *et al*. Identification of Viruses in Cases of Pediatric Acute Encephalitis and Encephalopathy Using Next-Generation Sequencing. *Sci. Rep*. **6**, 33452; doi: 10.1038/srep33452 (2016).

## Figures and Tables

**Table 1:** Summary of patients and virus detection with next-generation sequencing.

Patient no.	Age/Sex	Fever/Convulsions	Abnormality in brain imaging	Day specimens were obtained*	CSF cell count (/μl)	Virus detection (reads)/total reads			
						CSF		Serum	
						DNA sequencing	RNA sequencing	DNA sequencing	RNA sequencing
**1**	3 m/M	yes/no	no	0	379	ND	**Coxsackievirus A9** (2,783)/2,808,998	NA	NA
**2**	4 m/F	no/no	yes	1	65	none/8,083,731	none/4,042,588	**TTV (13)**/10,869,222	none/12,825,812
**3**	6 m/F	no/yes	yes	2	2	ND	none^†^/9,631,224	none/14,029,721	none/13,057,234
**4**	1 y/F	yes/yes	yes	0	2	ND	none^†^/6,126,780	none/7,511,583	none/6,503,784
**5**	1 y/F	yes/yes	no	1	42	ND	none^†^/9,409,404	none/8,761,917	none/9,382,070
**6**	1 y/F	yes/yes	no	2	2	NA	NA	none/5,534,632	none/9,901,028
**7**	2 y/F	yes/yes	no	0	175	none/7,986,561	none/3,729,914	NA	NA
**8**	2 y/M	yes/yes	yes	1	1	ND	none^†^/10,469,992	none/8,551,602	none/16,207,392
**9**	3 y/M	yes/no	no	0	151	ND	**Mumps virus (1,598)**/6,342,942	ND	none/9,858894
**10**	3 y/M	yes/yes	no	1	3	ND	none^†^/9,903,654	none/10,173,039	none/7,157,566
**11**	4 y/M	yes/yes	yes	1	2	NA	NA	none/6,160,773	none/8,588,056
**12**	7 y/M	yes/no	no	2	25	ND	**Coxsackievirus A9 (316)**/5,017,612	NA	NA
**13**	8 y/M	yes/yes	yes	0	2	ND	none^†^/10,693,530	none/5,161,481	none/8,437,476
**14**	10 y/F	yes/yes	no	2	1	ND	none^†^/21,903,050	none/6,165,412	**Pepper mild mottle virus (298)**/10,001,178
**15**	12 y/F	yes/yes	no	2	1	ND	none^†^/5,319,1116	none/2,790,344	none/9,769,956
**16**	13 y/M	yes/no	yes	2	1044	none/11,177,479	none/4,416,258	none/9,583,470	none/9,092,904
**17**	14 y/M	yes/yes	yes	1	162	none/4,037,654	none/2,229,247	none/26,686,548	none/6,830,980
**18**	15 y/M	yes/no	no	1	2	ND	none^†^/7,971,760	none/6,818,809	none/9,851,214

Metagenome analysis for virus identification was performed using the MePIC pipeline. The number of reads mapping to viral sequences was analyzed with the CLC workbench.

CSF, cerebrospinal fluid; ND, not done; NA, samples not available; TTV, Torque teno virus; *, days after onset of illness; ^†^, RNA was extracted using carrier RNA.

**Table 2:** Primers and probe used for virus detection.

Virus (methods)	Primer/Probe	Sequence*	Position
Human herpesvirus 6 (real-time PCR)	Forward	TTTGCAGTCATCACGATCGG	46661−46680
	Reverse	AGAGCGACAAATTGGAGGTTTC	46862−46883
	Probe	Cy5-AGCCACAGCAGCCATCTACATCTGTCAA-BHQ3a	46753−46780
Coxsackievirus A9 (RT-nested PCR)	Forward-outer	GTTGAGACAGGACAYACGTC	2526–2545
	Reverse-outer	CTRATRAATGGGATTGACATGC	2986–2965
	Forward-inner	GAYACYATGCAGACYAGGCACGT	2562–2584
	Reverse-inner	GYGCGTTYCCCTCTGTCCA	2958–2940
Mumps virus (RT-nested PCR)	Forward-outer	GCRACYAAAGARATCAGRAGRATC	6076–6099
	Reverse-outer	AGCCTTGATCATTGATCATCC	6803–6783
	Forward-inner	TCAAGYAGTGTCGAYGATCTC	6130–6150
	Reverse-inner	TGTCAGCCGCATTGATAACAGG	6689–6668
Pepper mild mottle virus (RT-PCR)	Forward	ACATTTGGACGACGCTGT	4778–4805
	Reverse	CGAGTTCTGCCCAATTCTAAC	5649–5629

^*^Sequences are shown 5′ to 3′ with standard codes (Y = C/T, R = A/G).
